# Comparison of three different ELISAs for the detection of recombinant, native and plasma IL-17A

**DOI:** 10.1016/j.mex.2020.100997

**Published:** 2020-07-16

**Authors:** Mohamad Bachar Ismail, Selma Olsson Åkefeldt, Magda Lourda, Désirée Gavhed, Rémi Gayet, Maurizio Aricò, Jan-Inge Henter, Christine Delprat, Hélène Valentin

**Affiliations:** a Laboratoire Microbiologie Santé et Environnement, Doctoral School of Sciences and Technology, Faculty of Public Health, Lebanese University, Tripoli, Lebanon; b Childhood Cancer Research Unit, Department of Women's and Children's Health, Karolinska Institutet, Karolinska University Hospital, Stockholm, Sweden; c Theme of Children's and Women's Health, Karolinska University Hospital, Stockholm, Sweden; d Center for Infectious Medicine, Department of Medicine, Karolinska Institutet, Stockholm, Sweden; e Faculté de Médecine, Groupe sur l'Immunité des Muqueuses et Agents Pathogènes (GIMAP), 10 rue de la Marandière, 42270 Saint Priest en Jarez, France; f Azienda Ospedaliero-Universitaria A. Meyer Children Hospital, Firenze, Italy; g UnivLyon, Université Claude Bernard Lyon 1, Villeurbanne, France; h INSERM U1052, CNRS UMR5686, Cancer Research Center of Lyon (CRCL), Lyon, France; i Ospedale Pediatrico Giovanni XXIII Azienda Ospedaliero Universitaria Consorziale Policlinico Bari

**Keywords:** ELISA, Interleukin-17A, 500-P07G antibody, 41802 antibody, eBio64CAP17 antibody, Langerhans Cell Histiocytosis, Plasma, Inflammation, IL-17A, Interleukin-17A, LCH, Langerhans Cell Histiocytosis, PBMCs, peripheral blood mononuclear cells, PBLs, Peripheral Blood Lymphocytes, ELISA, Enzyme-Linked-Immunosorbent-Assay, Ab, antibody, mAb, monoclonal Antibody, OD, optical density, E_500-P07G, ELISA using 500-P07G capture polyclonal Ab, E_41802, ELISA using 41802 capture mAb, E_eBio64CAP17, ELISA using eBio64CAP17 capture neutralizing mAb, rhIL-17A, recombinant human IL-17A, CI, Confidence Interval

## Abstract

Plasma IL-17A detection in Langerhans Cell Histiocytosis (LCH) is currently a source of debate. Indeed, 500-P07G (PeproTech) and 41802 (R&D Systems) anti-IL-17A antibodies have been suspected to recognize nonspecific proteins. To resolve this discrepancy, we set up two new ELISAs by using 41802 or neutralizing eBio64CAP17 (eBioscience) capture monoclonal antibodies that we compared to the commercial PeproTech ELISA kit. The three ELISAs, called E_500-P07G, E_41802 and E_eBio64CAP17, differ in their anti-IL-17A capture antibodies: either polyclonal, monoclonal or neutralizing monoclonal antibodies, respectively. Here, we show that these ELISAs had a similar capacity to specifically detect recombinant or native human IL-17A. However, a significantly lower plasma IL-17A detection was obtained with E_41802 compared to the two other ELISAs. Both E_500-P07G and E_eBio64CAP17 showed similar results. Consequently, we propose that the use of E_500-P07G and E_eBio64CAP17 may ensure more accurate and reliable results in the context of LCH studies. The highest plasma IL-17A levels in LCH patients compared to controls detected by both E_500-P07G and E_eBio64CAP17 ELISAs led us to propose these latter as reference techniques to investigate IL-17A as a potential new biomarker in LCH.

• The customization of a new E_eBio64CAP17 ELISA is suitable to detect human IL-17A.
• E_eBio64CAP17 ELISA protocol differs only in the anti-IL-17A capture antibody compared to the commercial E_500-P07G PeproTech kit.
• Data generated using the E_eBio64CAP17 ELISA are consistent with the PeproTech kit.

## Graphical abstract

**Figure:**
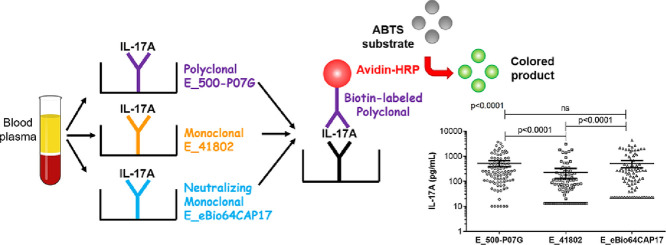

**Specifications table**

**Table:** 

Subject Area	Immunology and Microbiology
More specific subject area	Plasma IL-17A quantification by new Enzyme-Linked-Immunosorbent-Assays (ELISAs*)*
Method name	ELISA
Name and reference of original method	Human IL-17A ELISA development Kit from PeproTech (# 900-K84 catalog)
Resource availability	

## Method details

### Background

Interleukin-17A (IL-17A) is a major pro-inflammatory cytokine essential in immune responses. However, IL-17A also contributes to the development of several human chronic inflammatory diseases [1], [2], [3]. In Langerhans Cell Histiocytosis (LCH), a rare granulomatous disease, the detection of IL-17A in plasma of patients is still a matter of debate. A higher level of plasma IL-17A in LCH patients compared to healthy controls was reported by four laboratories, while another failed to demonstrate it or only to a very low level [4], [5], [6], [7], [8], [9], [10]. The origin of this apparent discrepancy is the use of different anti-IL-17A antibodies (Abs). Indeed, 500-P07G PeproTech polyclonal Ab and 41802 R&D Systems monoclonal Ab (mAb) have been ascribed to recognize nonspecific proteins different from IL-17A [9]. To overcome the issue of IL-17A detection in LCH, we set up two new ELISAs using 41802 or eBio64CAP17 (eBioscience) capture mAbs; the latter being a neutralizing Ab, i.e. highly specific. In order to solve these discordant results, we therefore compared these two new ELISAs to the PeproTech Development Kit ELISA, which has been used in several studies for the detection of IL-17A in the plasma of LCH patients.

### Characteristics of antibodies used

The data sheet of the polyclonal goat anti-IL-17A (# 500-P07G) from PeproTech indicates that this Ab is used in sandwich ELISA (ELISA Kit # 900-K84, PeproTech). 500-P07G polyclonal Ab is used as the capture Ab at a concentration of 0.5 µg/mL in this ELISA in combination with the biotinylated 500-P07G Ab (PeproTech, 0.25 µg/mL) for recombinant human IL-17A (rhIL-17A) detection. It is not a neutralizing Ab. The reactivity of this polyclonal Ab with other IL-17 family members is not mentioned in the data sheet.

The data sheet of the monoclonal mouse IgG1 anti-IL-17A (clone # 41802) from R&D Systems indicates that this mAb detects human IL-17 in direct ELISAs, but, is not a neutralizing Ab and has never been described in a sandwich ELISA. No cross-reactivity with rhIL-17E or rhIL-17F was observed by R&D Systems in direct ELISA.

The data sheet of the neutralizing monoclonal mouse IgG1 anti-IL-17A (clone # eBio64CAP17) from eBioscience indicates that this mAb detects human IL-17A in sandwich ELISA. eBio64CAP17 mAb is used as the capture antibody in ELISA in combination with the biotinylated eBio64DEC17 Ab (eBioscience) for rhIL-17A detection. The suitable concentrations range of eBio64CAP17 mAb for ELISA capture is 0.5 to 2.0 µg/mL. The reactivity of the eBio64CAP17 mAb with other IL-17 family members has not been evaluated.

### Blood samples and activation of lymphocytes from peripheral blood lymphocyte

Plasma and peripheral blood mononuclear cells from healthy volonteers and LCH patients were isolated after Ficoll gradient separation. Then, plasma samples were stored at −20 °C. Peripheral blood lymphocytes (PBLs) from healthy volunteers were collected from fresh peripheral blood mononuclear after Percoll gradient separation and then cultured in complete RPMI culture medium supplemented with 10% FCS. Native IL-17A from culture supernatants was obtained after stimulation of PBLs by Ionomycin (1 µg/mL) and Phorbol 12-Myristate 13-Acetate (10 ng/mL) for three days.

Upon approval by their institutional review boards, proven LCH patients were enrolled by the two major clinical centers with expertise in LCH (Karolinska University Hospital, Stockholm, Sweden and Azienda di Rilievo Nazionale ed Alta Specializzazione Civico-Ospedale dei Bambini, Palermo, Italy). In this study, adults with pulmonary LCH were excluded. As controls, healthy donors from Etablissement Français du Sang (France) and Karolinska University Hospital (Stockholm, Sweden) were recruited. At sampling, controls had no viral, inflammatory and malignant disease. For controls and LCH patients, a clinical questionnaire and written informed consent were obtained.

### Required reagents and equipment for Enzyme-Linked-Immunosorbent-Assay (ELISA)

• Tween-20
• BSA
• Dulbecco's PBS-10X
• rhIL-17A, rhIL-17F and rhIL-17E (PeproTech)
• Capture Abs: 500-P07G, eBio64CAP17 or 41802 anti-IL-17A
• Detection Ab: biotinylated 500-P07G (PeproTech) anti-IL-17A
• Avidin peroxidase conjugate (Avidin-HRP, PeproTech)
• ABTS liquid substrate solution
• ELISA microplates
• ELISA plate reader
• Blocking buffer: 1% BSA in PBS-1X
• Washing buffer: 0.05% Tween-20 in PBS-1X
• Dilution buffer: 0.05% Tween-20, 1% BSA in PBS-1X

### Procedure for IL-17A detection using three different ELISAs

The concentration of native and plasma IL-17A was quantified using the commercial sandwich ELISA kit (# 500-P07G from PeproTech) according to the manufacturer's instructions. This ELISA was used as reference and compared to the two other homemade ELISAs, which only differed from the PeproTech ELISA by the capture Ab used. The three ELISAs used in this study are thus abbreviated by the name of the capture Ab, namely E_500-P07G, E_41802 and E_eBio64CAP17.

1. For the adsorption of capture Ab, dilute 500-P07G, 41802 and eBio64CAP17 with PBS-1X at a final concentration of 0.5 µg/mL, and 4 µg/mL, respectively. Then, add 100 µL of each of the Abs to each well and cover the ELISA plate.
2. Incubate overnight at room temperature. The next steps are similar to the commercially available E_500-P07G:
3. Aspirate the wells to remove the liquid and wash the plate four times using 250 µL of washing buffer per well. After the last wash invert plate to remove residual buffer and blot on paper towel.
4. For saturation, add 300 µL blocking buffer to each well and cover the ELISA plate. Incubate for at least 1 h at room temperature.
5. Aspirate and wash plate four times as described in step 3.
6. Dilute standard rhIL-17A from 2 ng/mL to zero in dilution buffer. Then, add 100 µL of rhIL-17A or sample to each well in duplicate and cover the ELISA plate.
7. Incubate at room temperature for at least 2 h.
8. Aspirate and wash plate four times as described in step 3.
9. For detection, dilute biotinylated 500-P07G Ab in dilution buffer at a final concentration of 0.25 µg/mL. Then, add 100 µL per well and cover the ELISA plate.
10. Incubate at room temperature for 2 h.
11. Aspirate and wash plate four times as described in step 3.
12. Dilute avidin-HRP 1:2000 in dilution buffer. Then, add 100 µL per well and cover the ELISA plate.
13. Incubate 30 min at room temperature.
14. Aspirate and wash plate four times as described in step 3. Aspirate all the bubbles.
15. Add 100 µL of ABTS substrate solution, which should be at room temperature prior to use, to each well without making bubbles. Cover the ELISA plate.
16. Incubate at room temperature in the dark for color development.
17. Read the plate with an ELISA plate reader at 405 nm (possibly with wavelength correction set at 650 nm) at five-minute intervals for 15 min. The optical density (OD) does not exceed 1.2 or 1.4 depending on the batch.
18. A standard rhIL-17A curve was drawn using grade 4 polynomial regression, and OD values were then interpolated to determine sample IL-17A concentrations.
19. The limit of detection for E_500-P07G, E_41802 and E_eBio64CAP17 were 10, 13 and 23 pg/mL, respectively, which are the threshold values.

### Statistical analysis

The non-parametric Mann–Whitney U test was carried out to compare plasma IL-17A from healthy volunteers and LCH patients. For the comparison of three ELISAs, two tests were used: the non-parametric Kruskal–Wallis test with Steel-Dwass-Critchlow-Fligner procedure or the paired t-test with the Welch-Satterthwaite formula. P values smaller than 0.05 were accepted as statistically significant. For statistical analyses, the XLSTAT-Biomed module software (Version 19.6, Addinsoft) was used, while the graphs were created using GraphPad Prism software (Version 5.0, GraphPad Software Inc.).

## Method validation

The association between IL-17A and LCH has been the subject of debate due partially to discordant results. In this study, we aimed at clarifying the issue by acting on the technical study design. Here, we compared three IL-17A ELISAs that differ in the characteristics of their anti-IL-17A capture Ab. Indeed, 500-P07G, 41802 and eBio64CAP17 are a polyclonal, a monoclonal and a neutralizing monoclonal anti-17A Abs, respectively.

**Fig. 1:**
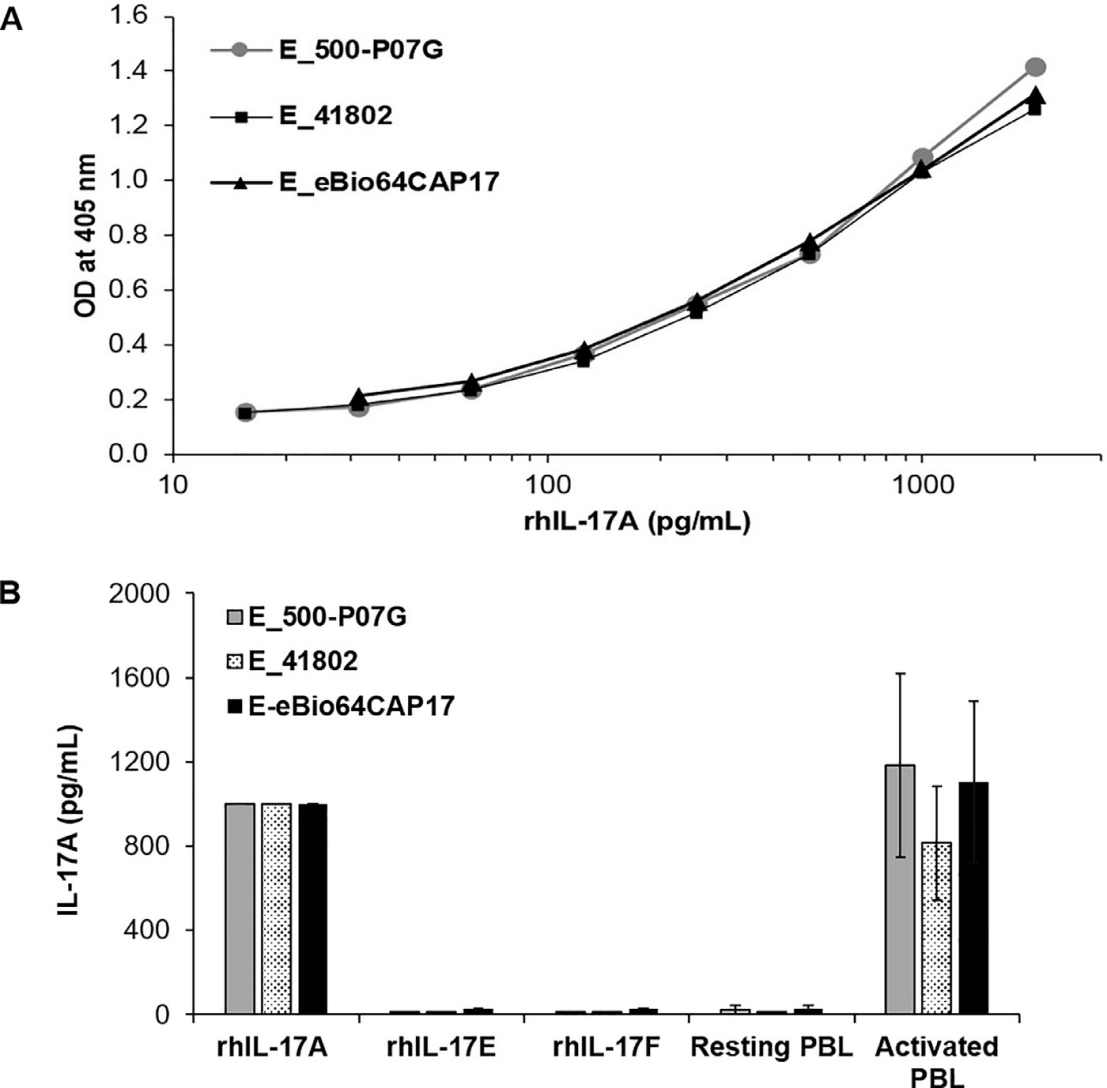
Comparison of E_500-P07G, E_41802 and E_eBio64CAP17 for the detection of recombinant and native human IL-17A. (A) Measurement of rhIL-17A standard range, using either the E_500-P07G or E_41802 or E_eBio64CAP17 ELISA, corresponds to the mean of three independent experiments. SD < 10%. (B) Detection of rhIL-17A (1000 pg/mL), rhIL-17E (2000 pg/mL), rhIL-17F (2000 pg/mL) or native IL-17A from PBLs (mean ± *S*.D, *n* = 4) by either E_500-P07G or E_41802 or E_eBio64CAP17 ELISA. Supernatants from resting or activated PBLs from four independent donors were evaluated after three days of culture.

We first compared the detection of human recombinant and native IL-17A (Fig. 1). Measurement of a rhIL-17A standard range demonstrated that the exponential and saturation phases of E_500-P07G, E_41802 and E_eBio64CAP17 ELISAs were similar, while the threshold of detection was 10 pg/mL, 13 pg/mL and 23 pg/mL, respectively (Fig. 1A). For the three ELISAs, the specificity for rhIL-17A was demonstrated by the absence of signal with 2000 pg/mL of rhIL-17E or rhIL-17F (Fig. 1B). As expected, the three ELISAs did not detect IL-17A in the supernatant of resting PBLs. Native hIL-17A, produced by activated PBLs, was similarly detected by the three ELISAs, although E_41802 appeared to be less efficient in the detection of native IL-17A compared to the two other ELISAs (Fig. 1B).

**Fig. 2:**
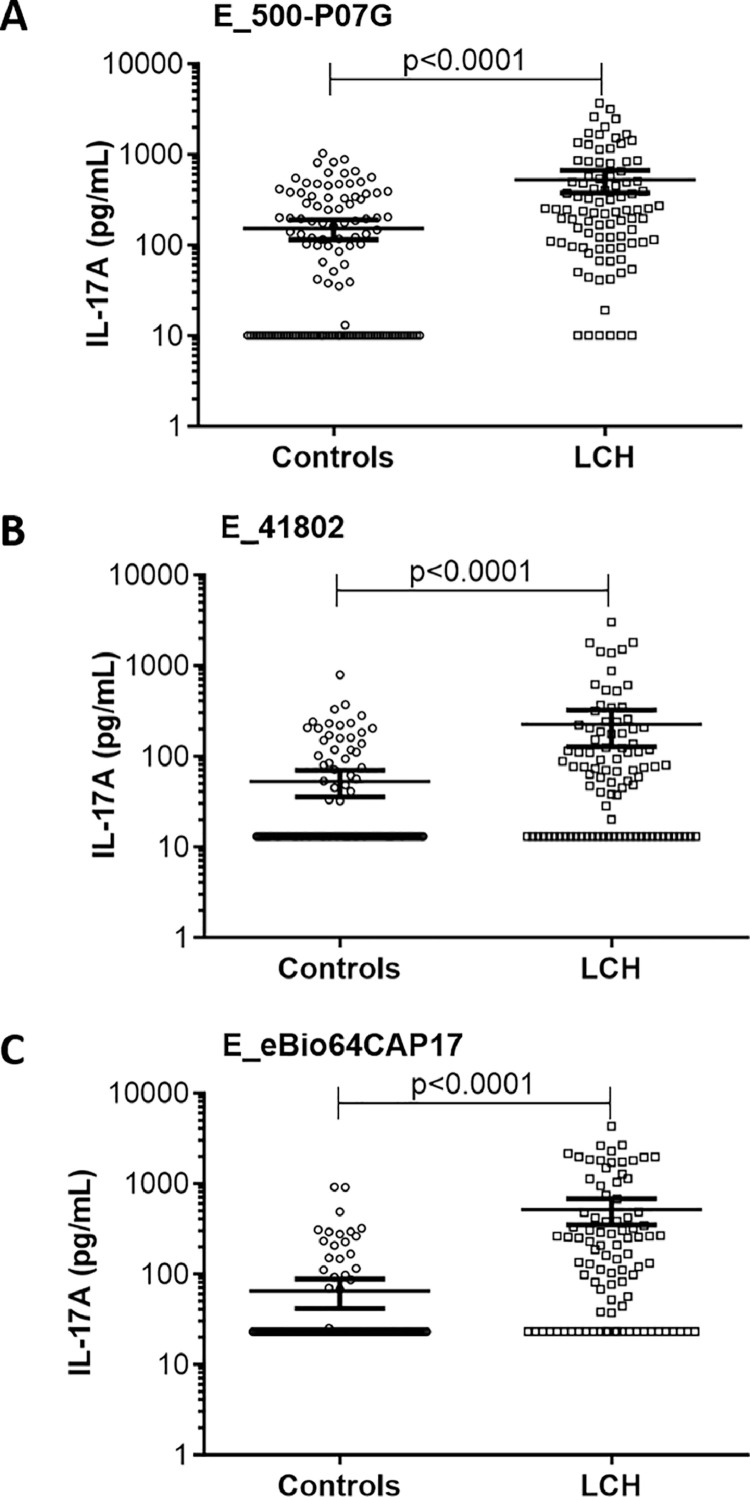
Quantification of plasma IL-17A levels in controls (*n* = 127) and LCH samples (*n* = 92) using either the E_500-P07G or E_41802 or E_eBio64CAP17 ELISA. The results are displayed in scatter plots with individual plasma concentrations (pg/mL) in logarithmic scale. *p* values were calculated using the Mann-Whitney U test. Identical results were obtained using t-test with Welch's correction (not shown). Bars indicate the mean value ± 95% confidence intervals (CI).

To address the reported discrepancy, we analyzed plasma IL-17A levels in a larger cohort of LCH patients (92 samples from 68 patients) than in previous studies (which had included a number of patients ranging from 1 to 46). The mean value of IL-17A was significantly higher (*p* < 0.0001) in the 92 LCH samples than in the 127 controls by using either E_500-P07G (Fig. 2A) or E_41802 (Fig. 2B) or E_eBio64CAP17 (Fig. 2C). Therefore, regardless of the capture Abs used in ELISAs, the level of plasma IL-17A in LCH was higher than in controls.

**Fig. 3:**
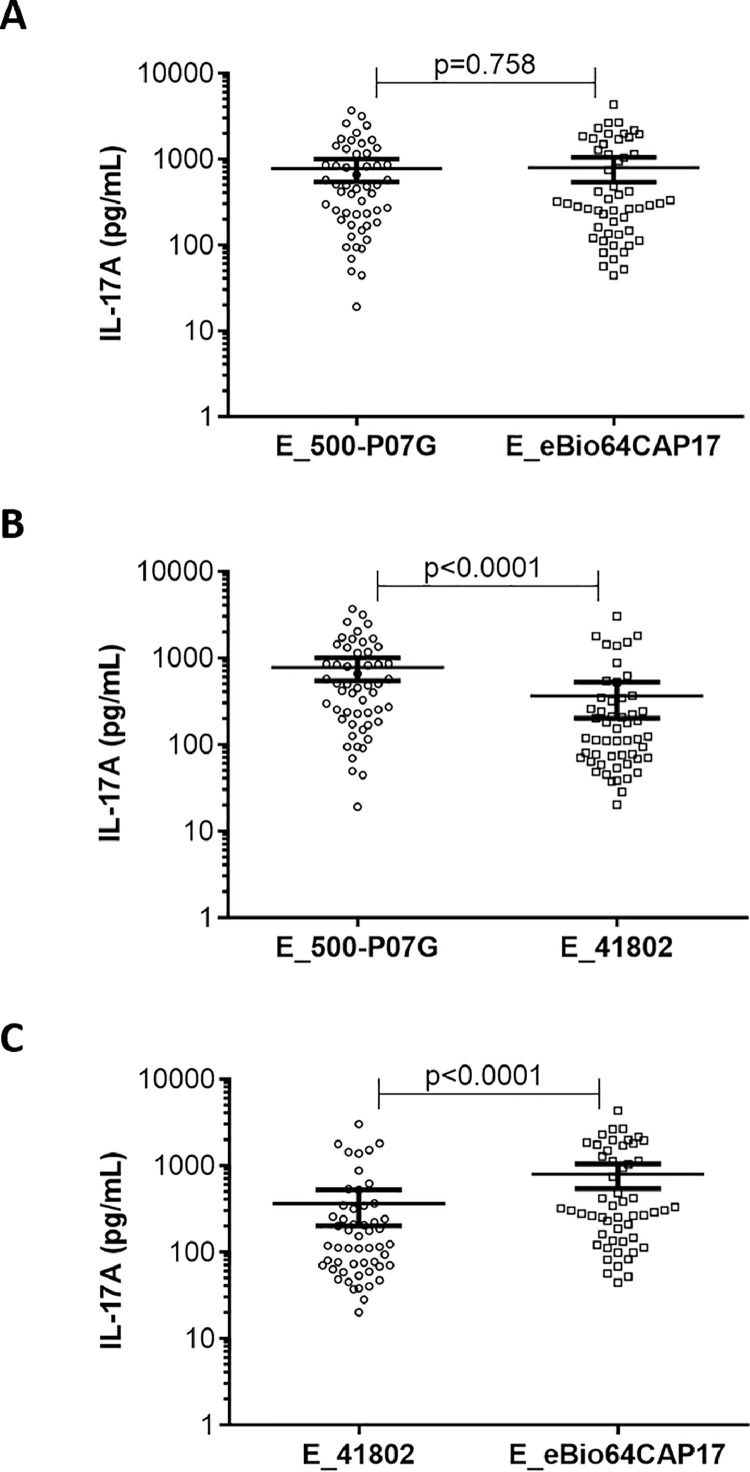
Paired comparison of positive plasma IL-17A from LCH samples (*n* = 53) detected by (A) E_500-P07G versus E_eBio64CAP17, (B) E_500-P07G versus E_41802, and (C) E_41802 versus E_eBio64CAP17. The results are displayed in scatter plots with individual plasma concentrations (pg/mL) in logarithmic scale. *p* value was calculated using paired t-test with the Welch-Satterthwaite formula. Bars indicate the mean with 95% CI.

Next, we determined whether the three ELISAs similarly recognized IL-17A in the plasma from LCH samples. To do that, we performed a paired comparison of positive plasma IL-17A from LCH samples (*n* = 53) for the three ELISAs. As shown in Fig. 3A, comparable IL-17A concentrations were detected by E_500-P07G and E_eBio64CAP17 in LCH plasma samples (*p* = 0.758). Surprisingly, this was not the case when we compared either E_500-P07G with E_41802 (Fig. 3B) or E_41802 with E_eBio64CAP17 (Fig. 3C) (*p* < 0.0001), suggesting that positive plasma IL-17 levels in LCH patients are more weakly detected by E_41802 compared to E_500-P07G and E_eBio64CAP17.

**Fig. 4:**
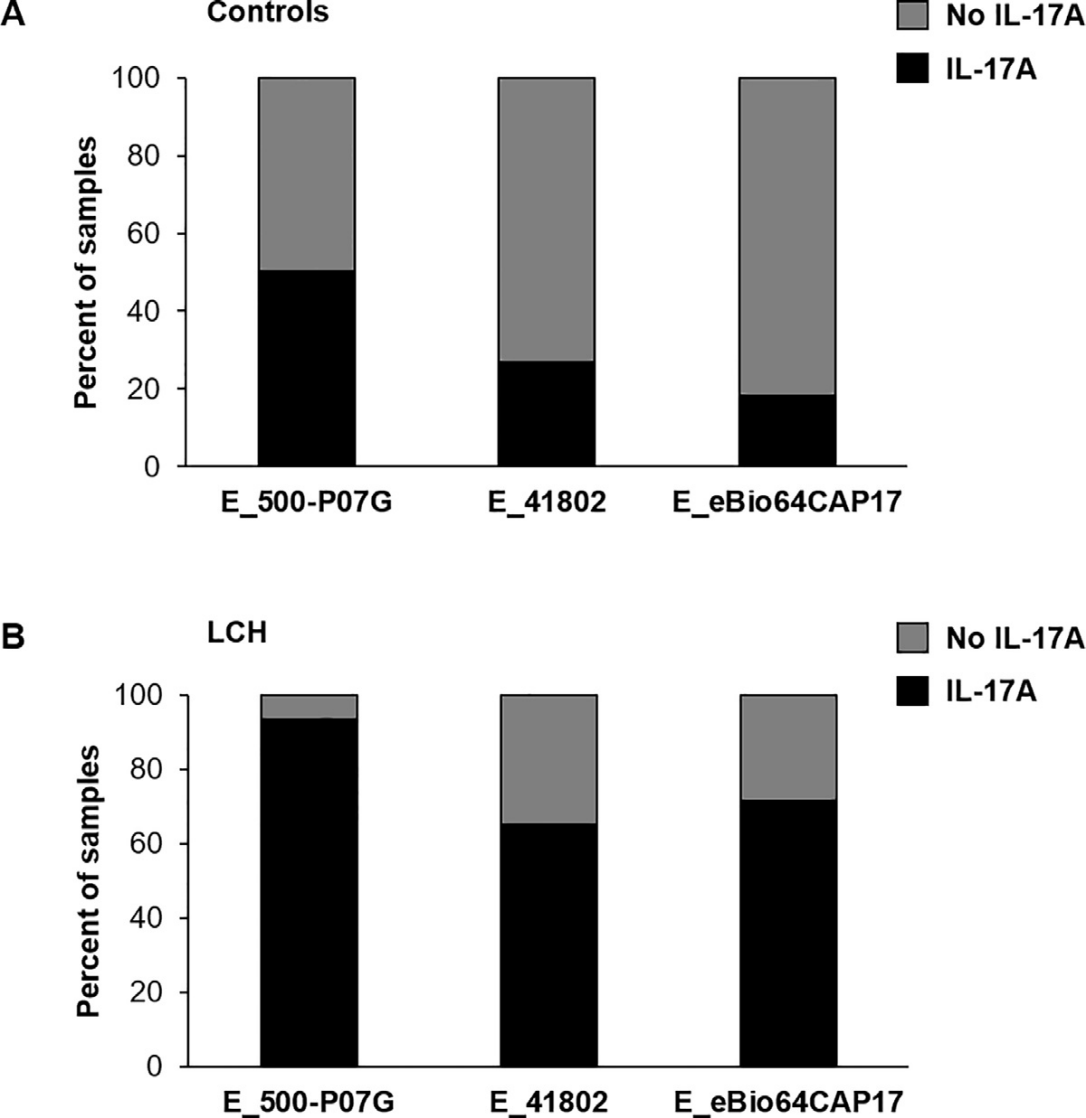
Comparison of E_500-P07G, E_41802 and E_eBio64CAP17 to assess the percentage of controls (A, *n* = 127) and LCH samples (B, *n* = 92) with detectable plasma IL-17A (black) or with no detectable IL-17A (gray). The threshold values are 10, 13 and 23 pg/mL for E_500-P07G, E_41802 and E_eBio64CAP17, respectively.

We then investigated to which extent E_41802 differed from the two other ELISAs. We analysed the percentage of plasma samples with no IL-17A detection and with IL-17A detection for the three ELISAs (Fig. 4). Regardless of the ELISAs used, the percentage of plasma samples with no IL-17A was higher than those with IL-17A detection in controls (Fig. 4A). Inversely, the percentage of plasma samples with IL-17A detection was higher than those with no IL-17A detection in LCH patients for the three ELISAs (Fig. 4B).

**Fig. 5:**
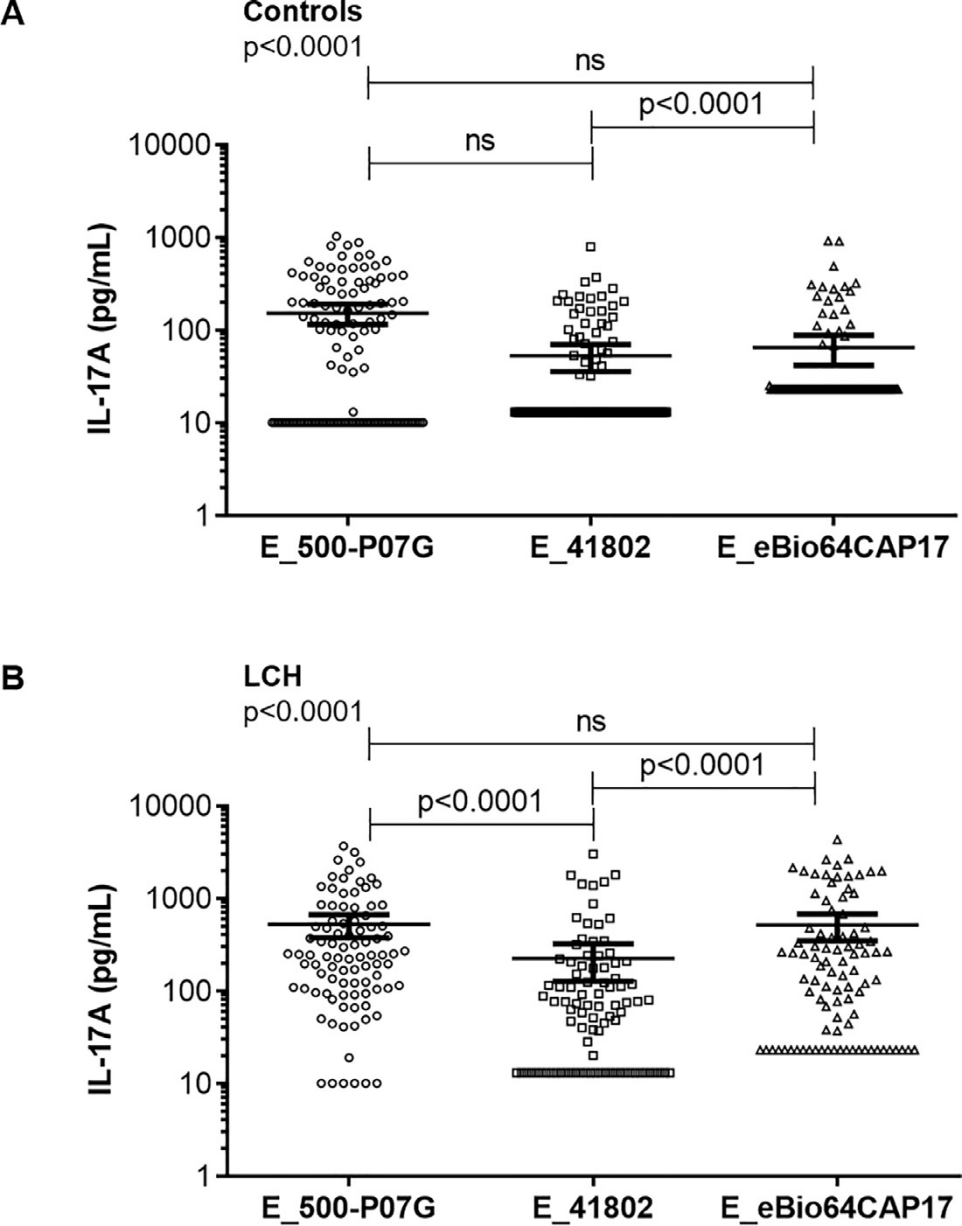
Comparison of the three ELISAs E_500-P07G, E_41802 and E_eBio64CAP17 for the quantification of plasma IL-17A levels in controls (A, *n* = 127) and LCH samples (B, *n* = 92). Data are presented as scatter plots with individual plasma IL-17A concentrations (pg/mL) in logarithmic scale. The Kruskal-Wallis test with Steel-Dwass-Critchlow-Fligner post-test were used to compare the groups and calculate *p* values. Bars indicate the mean value ± 95% CI. ns: not significant.

Finally, we compared the three ELISAs in term of their capacity to detect plasma IL-17A in controls (*n* = 127) on one hand and in LCH samples (92 samples from 68 patients) on the other hand (Fig. 5). We observed similar detection of plasma IL-17A in controls and in LCH samples using E_500-P07G and E_eBio64CAP17 (Fig. 5A-B). A difference was observed in the detection of plasma IL-17A between controls and LCH samples when using E_41802 and E_eBio64CAP17 (*p* < 0.0001) (Fig. 5A-B). Of note, E_500-P07G and E_41802 also differed for the detection of plasma IL-17 from LCH samples (*p* < 0.0001) (Fig. 5B).

## Discussion and conclusions

In our comparative study, we confirmed that the three E_500-P07G, E_41802 and E_eBio64CAP17 ELISAs detected comparable levels of both rhIL-17A and native IL-17A from activated PBL. However, the detection of human plasma IL-17A from LCH patients was more critical. Indeed, a significantly lower level of plasma IL-17A detection was evidenced using E_41802 compared to the two other ELISAs, while both E_500-P07G and E_eBio64CAP17 showed similar results. Thus, these latter tests may represent better tools to assess plasma IL-17A detection in LCH patients. One issue raised in the previous debate was the specificity of the polyclonal 500-P07G Ab. Among seven studies detecting plasma IL-17A from LCH patients using different procedures, three studies used the human IL-17A ELISA development kit from PeproTech as a reference [4,9,10]. Here, we demonstrate that the eBio64CAP17 neutralizing mAb and the 500-P07G polyclonal Ab are reliable to capture IL-17A in sandwich ELISAs. To our knowledge, our study is the first to show a comparison of three ELISAs to detect plasma IL-17A from a large cohort of LCH patients, reinforcing the presence of IL-17A in LCH. In addition to IL-17A, a storm of pro-inflammatory cytokines has been observed in the plasma of LCH patients, including IL-1 and IL-6 (see review [11]). The enhancing effect of IL-17A on IL-1-induced IL-6 was described several decades ago [12]. Its original mechanism has only recently been resolved: downstream of the IL-17A receptor, ACT1 targets a specific mRNA secondary structure of the IL-17A-regulated genes. Thus, IL-17A signaling potentiates the production of pro-inflammatory genes through the stabilization of their mRNA by ACT1 [13]. We investigated IL-6 content in 27 plasma LCH samples of our cohort and found 54% of positivity for IL-6 (Luminex assay, data not shown). Further studies in LCH lesions are required to confirm that IL-6 production is under the joint control of IL-1 transduction and ACT1-dependent ribonucleoprotein complexes induced by IL-17A.

The highest level of IL-17A detected in the plasma of patients with LCH compared to healthy controls suggested that IL-17A may be involved in some pathogenic mechanisms of LCH. In agreement with this hypothesis, a longitudinal study of a patient with progressive neurodegenerative LCH documented that high IL-17A levels in both blood and cerebrospinal fluid evolve with the disease progression [7]. These findings will be of interest to clinicians and researchers to further test IL-17A as a potential biomarker in samples of LCH patients by both E_500-P07G and E_eBio64CAP17 ELISAs, as we have previously suggested [10].
